# Smart Methylcellulose Hydrogels for pH-Triggered Delivery of Silver Nanoparticles

**DOI:** 10.3390/gels8050298

**Published:** 2022-05-12

**Authors:** Lorenzo Bonetti, Andrea Fiorati, Agnese D’Agostino, Carlo Maria Pelacani, Roberto Chiesa, Silvia Farè, Luigi De Nardo

**Affiliations:** 1 Department of Chemistry, Materials and Chemical Engineering “G. Natta”, Politecnico di Milano, Via Luigi Mancinelli 7, 20131 Milan, Italy; andrea.fiorati@polimi.it (A.F.); agnese.dagostino@polimi.it (A.D.); carlomaria.pelacani@mail.polimi.it (C.M.P.); roberto.chiesa@polimi.it (R.C.); silvia.fare@polimi.it (S.F.); luigi.denardo@polimi.it (L.D.N.); 2 National Interuniversity Consortium of Materials Science and Technology (INSTM), Via Giuseppe Giusti 9, 50121 Florence, Italy

**Keywords:** methylcellulose, citric acid, crosslinking, pH-responsive, silver nanoparticles (AgNPs)

## Abstract

Infection is a severe complication in chronic wounds, often leading to morbidity or mortality. Current treatments rely on dressings, which frequently contain silver as a broad-spectrum antibacterial agent, although improper dosing can result in severe side effects. This work proposes a novel methylcellulose (MC)-based hydrogel designed for the topical release of silver nanoparticles (AgNPs) via an intelligent mechanism activated by the pH variations in infected wounds. A preliminary optimization of the physicochemical and rheological properties of MC hydrogels allowed defining the optimal processing conditions in terms of crosslinker (citric acid) concentration, crosslinking time, and temperature. MC/AgNPs nanocomposite hydrogels were obtained via an in situ synthesis process, exploiting MC both as a capping and reducing agent. AgNPs with a 12.2 ± 2.8 nm diameter were obtained. MC hydrogels showed a dependence of the swelling and degradation behavior on both pH and temperature and a noteworthy pH-triggered release of AgNPs (release ~10 times higher at pH 12 than pH 4). ^1^H-NMR analysis revealed the role of alkaline hydrolysis of the ester bonds (i.e., crosslinks) in governing the pH-responsive behavior. Overall, MC/AgNPs hydrogels represent an innovative platform for the pH-triggered release of AgNPs in an alkaline milieu.

## 1. Introduction

Chronic wounds represent a severe clinical problem affecting 1–2% of the population in developed countries [1,2]. Being hard to heal, chronic wounds are challenging both for patients and healthcare systems, and account for 2–3% of the healthcare budgets [3]. According to clinical literature, chronic wounds can be assigned to three main categories: vascular ulcers, diabetic ulcers, and pressure ulcers [3]. Even if no single, widely accepted definition of chronic wound exists, in most cases, a wound is defined as chronic if it has not healed within a certain amount of time, ranging from 4 to 6 weeks for most authors, or up to 3 months, as noted in standard surgical textbooks [3,4]. Regardless of the time, chronic wounds share some standard features, e.g., persistent inflammation, recurrent infections with possible formation of antibiotic-resistant biofilms, and deficiency of stem cells. Combined, these pathophysiological phenomena result in the wound’s inability to heal [3].

Infection is a critical complication in chronic wounds, often leading to persistent nonhealing, significant morbidity, or even mortality. Alkalinity is associated with infection in chronic wounds, even if its cause is still unclear [5]. Bacteria metabolism could be responsible for the local increase of pH due to the release of ammonia and polyamines, which can impair the oxygenation of wound tissue and promote necrosis [6,7]. In this regard, several studies have shown how the pH of infected chronic wounds sometimes reaches values of 10 [5,6,8]. In addition, specific tissue-degrading enzymes (e.g., elastase, plasmin, matrix-metalloproteinase-2) display higher turnover rates in alkaline conditions. Consequently, an alkaline wound milieu may strongly contribute to tissue degradation and infection [6].

Topical wound therapies and dressings are primarily applied to treat chronic wounds: from standard cotton gauze dressings to highly absorbent and moisture-retaining foams, hydrocolloids, and hydrogels [9]. In many cases, due to the broad-spectrum antibacterial activity of silver, Ag-containing products provide an additional clinical option to manage bacterial growth [3,10,11]. However, silver misuse (e.g., uncontrolled release, excessive dosing) for a prolonged time can lead to potential toxicity to tissues and organs at the local and systemic levels [3,12].

Given the pH variations occurring in the wound milieu, stimuli-responsive polymers can take up the challenge of a smart (i.e., in response to pathophysiological phenomena) release of silver in high enough concentrations to ensure antibacterial activity [13]. Due to local pH variations, pH-responsive polymers undergo structural and property changes (e.g., dissolution/precipitation, degradation, swelling/collapsing, hydrophilic/hydrophobic change, change of shape, conformational change) [13,14]. The drop in pH has been investigated for the controlled release of silver nanoparticles (AgNPs) from different pH-responsive hydrogels (e.g., carboxymethyl chitosan/poly(vinyl alcohol), N-acryloyl-N′-ethyl piperazine/N-isopropylacrylamide) [15,16]. However, only a few studies reported the possibility of exploiting the alkalinity of infected wounds to trigger the release of AgNPs [12].

In this study, methylcellulose (MC) hydrogels were prepared and crosslinked with citric acid (CA) at three different crosslinking degrees: low (MC-L), medium (MC-M), and high (MC-H). Pristine hydrogel (MC) was used as a control. The thermo- and pH-responsiveness of the samples were assessed by swelling/degradation and rheological tests at different pH (4, 7, 12) and temperatures (25, 37, 50 °C). MC-H samples were further investigated due to their remarkable pH-responsive behavior. ^1^H-NMR spectrometry was exploited to disclose the mechanism of pH-responsiveness. Afterward, MC–H/AgNPs nanocomposite hydrogels were prepared via AgNPs in situ synthesis using MC both as a capping agent and reducing agent. TEM and UV–vis measurements assessed the shape, size, and distribution of AgNPs. Lastly, ICP and UV–vis measurements supported a quantitative evaluation of the pH-triggered AgNPs release mechanisms to develop systems with enhanced antibacterial activity in alkaline conditions.

## 2. Results and Discussion

### 2.1. Swelling and Degradation Tests

Swelling tests were carried out in normal saline solution (NSS) adjusted to different pH levels and at different temperatures to evaluate the pH-and thermo-responsiveness of MC hydrogels. Due to the lack of buffering ability of NSS, the solution was refreshed every 72 h to preserve the pH around the desired value. In this first part of the study, NSS (0.15 M NaCl) was selected to minimize the influence of the salt on the T_t_. A low NaCl concentration (i.e., ~0.1 M) has been reported to affect MC gelation properties slightly. Conversely, other salts (e.g., phosphates, sulfates), strongly acting on the interactions between MC chains and water molecules, have been reported to affect the T_t_ of MC significantly even at low concentrations (i.e., <0.2 M) [17,18].

**Figure 1:**
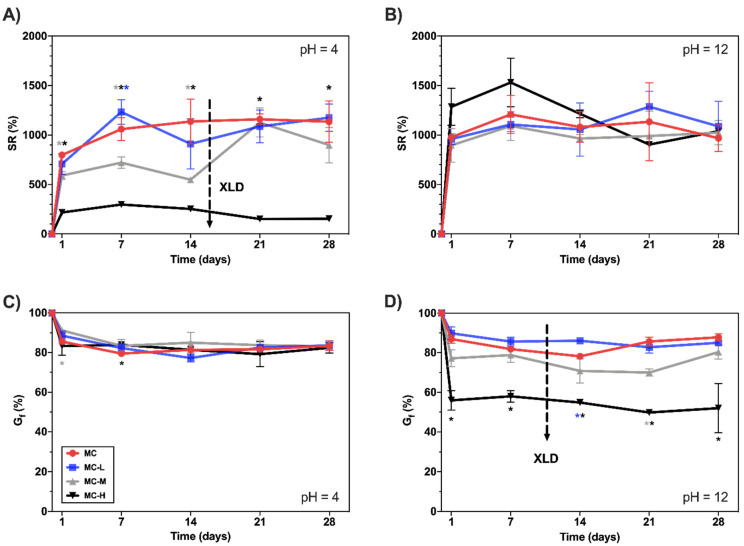
SR (%) vs. time of MC hydrogels in NSS at T = 37 °C. (**A**) pH = 4; (**B**) pH = 12. G_f_ (%) vs. time curves of MC hydrogels samples in NSS at T = 37 °C. (**C**) pH = 4; (**D**) pH = 12. * = *p* < 0.05 compared to MC control (* = MC vs. MC-L, * = MC vs. MC-M, * = MC vs. MC-H).

Figure 1 reports the swelling curves for the MC specimens at 37 °C. For clarity, Figure 1 only reports the curves at pH = 4 and 12. (Refer to Supplementary Materials Figure S1 for pH = 7.) At pH 4 (Figure 1A), a decrease in the SR (%) as a function of the crosslinking degree (XLD, black arrow) can be observed. This trend agrees with previous data on the same hydrogel formulation [19,20], even if lower SR values are observed, probably due to the different swelling solutions (NSS vs. distilled water). At pH = 7 (Supplementary Materials Figure S1), the curves retrace the ones at pH = 4. At pH = 12 (Figure 1B), all the specimens reach a swelling plateau between days 1 and 7, and display no significant differences (*p* > 0.05) despite their different crosslinking degree.

Gel fraction tests were then performed to evaluate the dissolution of MC hydrogels as a function of the pH of the NSS. At pH = 4 (Figure 1C), no differences (*p* > 0.05) among MC samples can be observed. All the samples reach a plateau, with G_f_ = 80% within the first week of the test. Such results are slightly discordant with previous outcomes on the same hydrogel formulations where higher degradation was assessed on MC samples [19]. As for swelling tests, the overall reduced degradation in this study can be attributed to the different swelling media (NSS vs. distilled water). Cl^-^ anions, destabilizing water–polymer interactions, lead to the association of MC hydrophobic groups, increasing the G_f_ values [21]. At pH = 7 (Supplementary Materials Figure S1), the curves retrace the ones at pH = 4. At pH = 12 (Figure 1D), it is possible to distinguish a trend in the G_f_ values as a function of the XLD of the samples. A decrease in the G_f_ values is associated with higher crosslinking rates (G_f_ = 80% vs. 55% at day 7 for MC and MC-H specimens, respectively).

These findings support what was obtained with the swelling tests (Figure 1B), where in the same conditions (pH = 12, T = 37 °C), the highly crosslinked sample swelled more than at lower pH. Thus, it is possible to suppose that the increased swelling (and reduced G_f_) is due to the occurrence of two concurrent phenomena: (i) the deprotonation of the free -COOH groups in the MC-H samples, which leads to electrostatic repulsion that, in turn, increases the pore size of the hydrogel network [12]; (ii) the alkaline hydrolysis of the ester groups in the MC-H sample [19,22]. These phenomena lead to the disruption of the MC-H hydrogel network, with a resultant increase in swelling and a loss of its physical stability.

**Figure 2:**
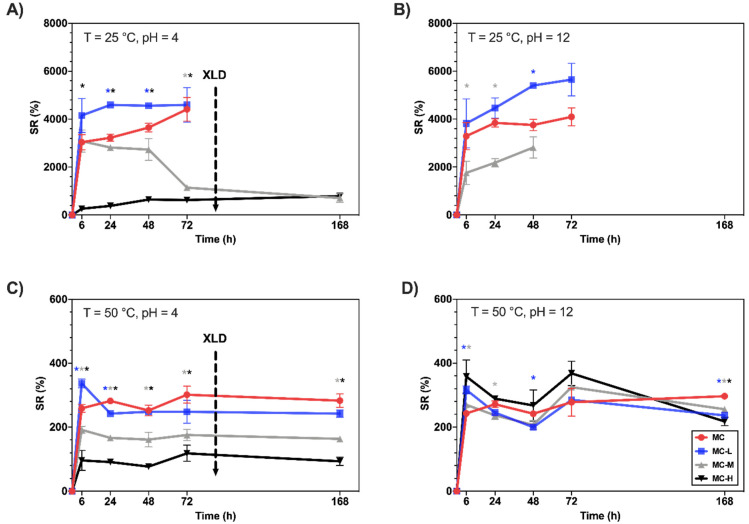
SR (%) vs. time of MC hydrogels in NSS at T = 25 °C and (**A**) pH = 4 or (**B**) pH = 12; at T = 50 °C and (**C**) pH = 4 or (**D**) pH = 12. * = *p* < 0.05 compared to MC control (* = MC vs. MC-L, * = MC vs. MC-M, * = MC vs. MC-H).

To further investigate the thermo-responsive behavior of crosslinked MC specimens, two additional swelling tests were conducted at T = 25 °C and T = 50 °C in NSS at different pHs. For clarity, Figure 2 only reports the curves at pH = 4 and 12 (refer to Supplementary Materials Figure S2 for pH = 7). As it is possible to observe in Figure 2A,B, at T = 25 °C, non-crosslinked and low-crosslinked specimens (MC, MC-L) underwent rapid swelling (up to SR = 4000–6000%), then dissolved within 72 h regardless of the pH of the NSS solution. In fact, at 25 °C (i.e., T < T_t_), MC was in a sol state and underwent fast dissolution in the water environment [19,20]. Conversely, more crosslinked specimens (MC-M and MC-H) display different behaviors according to the pH of the NSS. At pH = 4, MC-H specimens are stable until the end of the test (168 h). In contrast, at pH = 12, they undergo fast dissolution (induced both by alkaline hydrolysis of the ester groups and low temperature, i.e., T < T_t_) within the first 6 h of the test.

Different behavior can be observed at T = 50 °C (Figure 2C,D). First, lower SR values (SR = 100–400%) than those at T = 25 °C can be detected for both pH conditions, indicating a reduction of the swelling extent of all MC specimens induced by high temperature. This behavior occurs since, at 50 °C (i.e., T > T_t_), MC is in a gel state governed by inter- and intramolecular hydrophobic interactions between the methoxy groups present on its backbone. In this state, hydrogen bonds between water and the hydroxyl groups of MC are least favorable, leading to reduced swelling [19,21]. Interestingly, at pH = 4 (Figure 2C), a trend in swelling can still be observed as a function of the crosslinking rate, with swelling for pristine MC higher than for the high-crosslinked specimens (SR ~300% vs. ~100% for MC and MC-H, respectively). Conversely, at pH = 12 (Figure 2D), all the specimens behaved similarly, reaching SR values of ~300%.

For the first time, the possibility of recovering the thermo-responsive behavior of crosslinked MC hydrogels after alkaline hydrolysis of the ester bonds was demonstrated. Even though this is not further investigated in this work, the recovery of the thermo-responsiveness of crosslinked MC samples would open the floodgates to the development of dual-responsive (i.e., thermo- and pH-responsive) MC hydrogels, which can offer tremendous opportunities in several fields [13].

### 2.2. Rheological Characterization

**Figure 3:**
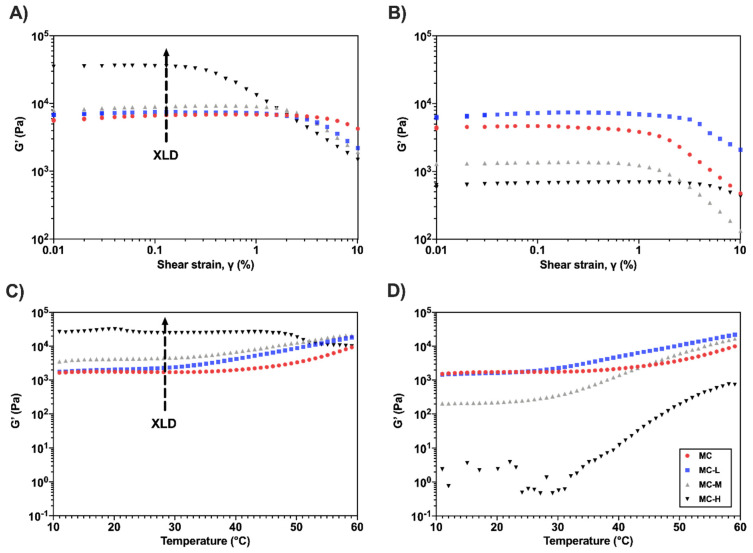
Representative G′ vs. γ curves for strain sweep tests on MC samples at (**A**) pH = 4 and (**B**) pH = 12. Representative G′ vs. T curves for temperature sweep tests on MC samples at (**C**) pH = 4 and (**D**) pH = 12.

The LVR region was identified qualitatively for all the hydrogels at γ < 0.1% via strain sweep tests. For clarity, Figure 3 only reports the curves at pH = 4 and 12. (Supplementary Materials Figure S3 reports the graphs at pH = 7.) Within the LVR (Figure 3A,B), the storage modulus is higher than the loss modulus (G′ > G″, data not shown) for each pH condition and each MC hydrogel type. This behavior suggests that the hydrogels are in a gel-like state, governed by weak interactions (i.e., R^1^-CH_3_····H_3_C-R^2^) and covalent bonds (i.e., ester bonds). Beyond the LVR, G′ linearity is lost, and the samples encounter a yielding phase, even if G′ > G″. Once the flow point (G′ = G″) is reached, the viscous component becomes preponderant, and the specimens start to flow [23,24]. The trend of the strain sweep curves in an acidic environment (Figure 3A) is similar to the results obtained in a previous work on the same hydrogel formulations [19]. As the crosslinking increases, the viscoelastic parameters increase. However, the different values of G′ and G″ here observed can be attributable to differences in the swelling medium (dH_2_O vs. NSS), denoting an increase in the mechanical properties accountable to a salting-out anion effect [21,25,26]. At pH = 7 (Supplementary Materials Figure S3) the curves retrace the ones at pH = 4. Conversely, different behavior can be observed at pH = 12 (Figure 3B): while the pristine and low crosslinked specimens (MC and MC-L) are unaffected by an external pH variation, an increase in crosslinking (MC-M and MC-H) results in a significant decrease in G′ (from 8 kPa to 1 kPa and from 30 kPa to 600 Pa for MC-M and MC-H, respectively). This change, most noticeable in the MC-H sample, is accountable for the alkaline hydrolysis of the ester bonds in the MC hydrogels network, as previously explained (Section 2.1). Despite the hydrolysis, all the hydrogel formulations maintained their gel-like state (G′ > G″) at pH = 12, suggesting that all the samples were above their T_t_ at the test temperature.

Temperature sweep tests were then conducted on all the hydrogel formulations (Figure 3C,D). At pH = 4 (and pH = 7, Supplementary Materials Figure S3), it is possible to observe an increase in G′ with the increase of the crosslinking degree, as previously reported [19]. A common trend was evident in all the temperature sweep curves (except for MC-H): (i) for T < T_t_, a slight decrease of G′ occurred due to the disentanglement of the MC chains; (ii) for T > T_t_, an abrupt increase of G′ occurred due to hydrophobic interaction between the MC chains (i.e., sol–gel transition) [27,28]. The MC-H specimen exhibited no sign of thermally triggering a sol–gel transition [19]. Conversely, at pH = 12, different behavior was observed. While the non-crosslinked and low crosslinked specimens (MC and MC-L) seem unaffected by the pH increase, the higher crosslinked specimens (MC-M and MC-H) displayed a significant decrease in G′. Interestingly, the MC-H samples seem to recover their temperature-induced sol–gel transition (Figure 3D), which was lost after chemical crosslinking (Figure 3C).

Table 1 reports the T_t_ values calculated by applying a previously reported method to the G′/T curves [28]. No significant difference (*p* > 0.05) was noticed between the T_t_ values of samples at the same pH (except for MC-L at pH = 4). This result suggests that the transition temperature was not affected by mild chemical crosslinking (MC-L and MC-M). Again, the lower T_t_ obtained in this work compared with the literature [19] is ascribable to the presence of salting-out anions in the NSS solution [21,25,26]. Interestingly, at pH = 12, MC-H displayed a T_t_~35 °C, which is in line with the other crosslinking conditions, confirming that the thermo-responsive behavior of this sample was recovered after hydrolysis (Section 2.1).

The higher crosslinking formulation (MC-H) was considered the most promising among the samples prepared for this work due to its remarkable pH responsiveness. Thus, all the subsequent characterizations were carried out only on MC-H samples.

### 2.3. ^1^H-NMR Characterization

**Figure 4:**
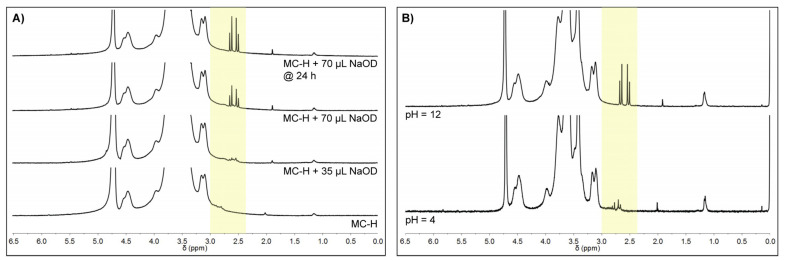
^1^H-NMR spectrum of (**A**) washed MC-H sample swelled in D_2_O and then hydrolyzed and (**B**) non-washed MC-H specimens swelled at different pH (4 vs. 12). All the spectra were acquired at 30 °C. The yellow area highlights the -CH_2_- peaks of CA.

The ^1^H-NMR spectra of MC-H samples are mainly characterized by the typical signals of MC [29]. The spectra also show -CH_2_- peaks in the spectral region between 2.5 and 3.0 ppm (Figure 4A, yellow), correlated with chemical crosslinking. Figure 4A reports an inset of the ^1^H-NMR spectra obtained on the washed MC-H samples. The bottom spectrum was obtained for MC-H at the swelling equilibrium (i.e., 24 h in D_2_O). A broad double doublet (dd) signal can be detected at 2.74–2.98 ppm: this set of signals can be attributed to the -CH_2_- hydrogens of CA covalently bonded to MC (ester bond). The top spectra were then obtained after adding definite amounts of NaOD. In particular, after the addition of 35 μL of NaOD, the previously detected broad signal shifts towards a higher field (2.60–2.85 ppm) [30], and a well resolved dd signal centered on 2.59 ppm (attributable to free CA) appears. These results suggest that the ester bonds between CA and MC can be easily hydrolyzed in strongly alkaline conditions. An additional 35 μL of NaOD resulted in a sensible increase in the intensity of the dd signals at 2.59 ppm and, at the same time, an appreciable reduction of the broad shoulders attributed to the covalently bonded CA. After 24 h, such broad shoulders are no longer detectable, confirming the hypothesis of the hydrolysis of the ester bonds in increasing alkaline conditions.

Figure 4B reports the ^1^H-NMR spectrum of non-washed MC-H specimens in NSS at different pH (i.e., same conditions of previous physical and rheological characterization). For samples at pH = 4, the observed dd signals at 2.75 ppm were attributed to unbonded (i.e., not washed off) CA. (Similar results have been obtained for pH = 7, Supplementary Materials Figure S4). A pH increase (pH = 12) led to a remarkable increase in the signal intensity accompanied by a shift to 2.59 ppm, confirming that ester bonds were completely hydrolyzed at this pH. These observations agree with swelling tests. In particular, swelling tests at pH = 12 (Figure 1B) revealed no significant differences (*p* > 0.05) between MC-H and MC specimens for the considered time points, supporting the fact that the complete hydrolysis of the ester bonds occurred at pH = 12 within 24 h.

In addition, by integrating the CA peaks before and after hydrolysis (pH = 4 vs. pH = 12), it was also possible to estimate that the content of unbonded CA in MC-H samples was about 10 mg g^−1^, and that the weight fraction of CA taking part in the ester linkage was equal to ~70%, which is in good accordance with previous findings [19].

**Figure 5:**
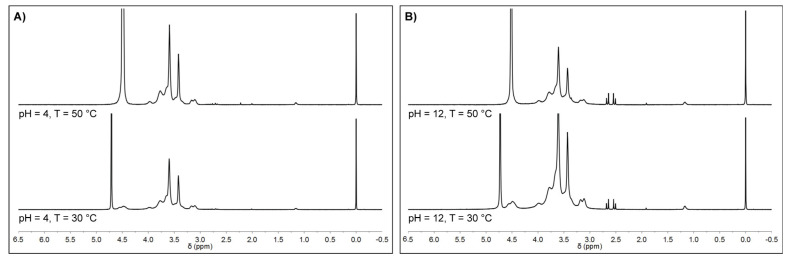
(**A**) ^1^H-NMR spectrum of MC-H swelled at (**A**) pH = 4 and (**B**) at pH = 12, as function of temperature (30 vs. 50 °C).

NMR spectra were then acquired as a function of temperature, and the overall signal intensity was evaluated to assess the thermally induced transition of MC-H specimens. Figure 5 reports the ^1^H-NMR spectrum of MC-H specimens as a function of temperature, at a swelling pH = 4 and 12. At pH = 4 (Figure 5A), the overall ^1^H-NMR signal intensity increased by increasing temperature. (Similar findings were obtained with samples at pH = 7, Supplementary Materials Figure S5). On the contrary, at pH = 12, the ^1^H-NMR signals decreased the intensity by increasing the temperature due to the restricted MC chains mobility induced by the sol–gel transition [31]. The thermally induced transition of MC to a more rigid and ordered structure caused a change in the spin–spin relaxation time (T_2_), leading to a sensible signal broadening [31]. These results confirm that the thermo-responsive behavior of the MC-H sample was retrieved after hydrolysis.

Overall, the results obtained for the MC-H samples open the floodgates to develop responsive hydrogels capable of undergoing pH-triggered, tailorable hydrolysis [32]. To the authors’ best knowledge, this is the first attempt at studying the pH-responsiveness of MC hydrogels. Similar systems have already demonstrated their potential in numerous biomedical applications [13,33], particularly for drug delivery (e.g., tumor-targeted drug delivery [34], intracellular delivery of nucleic acids or proteins [35], and the treatment of inflammatory diseases [36]). Considering this, the possibility of developing a novel pH-responsive platform based on MC for the delivery of AgNPs was investigated.

### 2.4. MC/AgNPs Composite Hydrogels

AgNPs were obtained by the chemical reduction of the metallic precursor (AgNO_3_) in the polymer matrix. Several methods have been reported in the literature to obtain AgNPs (e.g., citric acid reduction, electrochemical synthesis, photochemistry, and radiation reduction) [37]. In this work, MC acted both as a capping and reducing agent, combining the advantage of a one-step reaction with the possibility of a simple control over the reaction parameters (i.e., nanoparticle size and distribution) [37,38].

**Figure 6:**
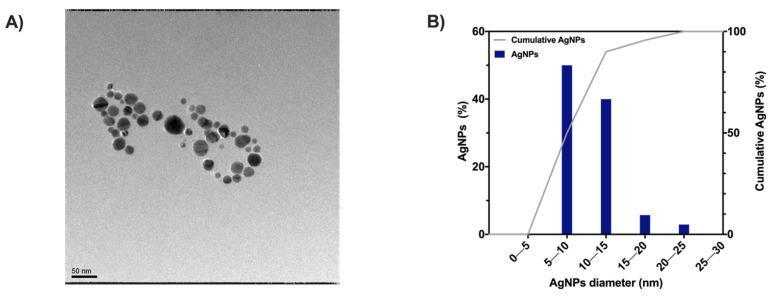
(**A**) TEM image of MC/AgNPs solution and (**B**) MC/AgNPs distribution analysis.

TEM observations provided insight into the shape and dimensions distribution of AgNPs (Figure 6A). The in situ synthesis approach allowed for obtaining spherical nanoparticles with 12.2 ± 2.8 nm diameter and a D90 of 15 nm (dimension distribution analysis in Figure 6B). The AgNPs here showed a slightly smaller size than those previously reported by Maity et al. [38] (mean diameter: 12.2 vs. 22 nm), which is likely due to the higher concentration of MC. Indeed, the concentration of capping and reducing agents plays an essential role in the size of silver nanoparticles [39,40]. Nevertheless, the obtained dimensions aligned with other works in which MC [41] or other polymers [37,42,43] have been reported for the in situ synthesis of metal nanoparticles. Moreover, the obtainment of AgNPs with smaller sizes (in the nanometric range) can show a non-trivial effect on their antibacterial activity efficiency due to their easy binding to important functional sites on pathogens [44,45,46].

### 2.5. MC-H/AgNPs Characterization: UV–Vis and ICP Analyses

**Figure 7:**
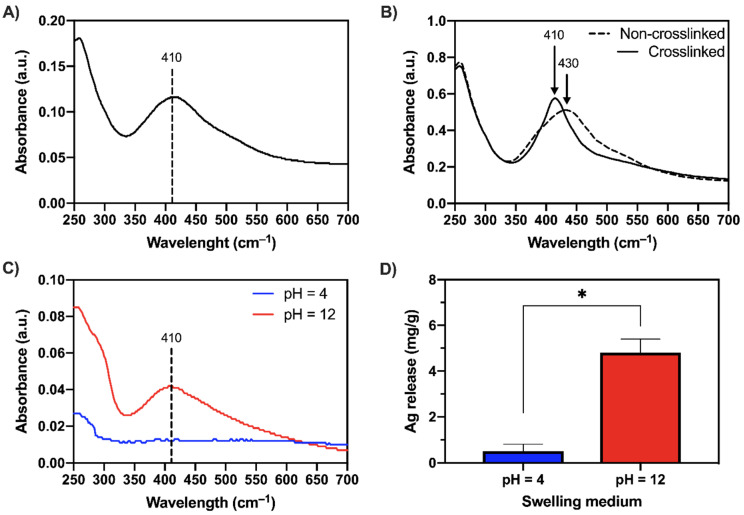
UV–vis absorption spectra of (**A**) as-synthesized MC/AgNPs, (**B**) MC-H/AgNPs dry films, (**C**) swelling media at pH = 4 and 12. (**D**) Ag release (mg_Ag_/g_MC_) from MC-H/AgNPs samples as a function of the pH obtained by ICP analyses. * = *p* < 0.05.

UV–vis analyses have been extensively reported in the literature to assess the formation and presence of AgNPs in suspension [44,45,46,47]. In this work, the as-prepared MC/AgNPs solutions (Figure 7A) displayed a well-defined absorption peak at 410 nm, which is typical of a colloidal suspension of spherical silver nanoparticles [47]. Such results are in accordance with previously reported works exploiting the dual role of MC as stabilizing and reducing agent for the in situ synthesis of AgNPs [38,41,48].

UV–vis measurements were then performed on MC-H/AgNPs specimens (in dry conditions, Figure 7B) to confirm the presence of AgNPs before and after crosslinking (190 °C, 15 min). For the non-crosslinked samples, a red shift (from 410 to 430 nm) occurs. This variation in the surface plasmon absorption peak position can be attributed to the different refractive index of the surrounding environment (i.e., dry film) [38,49]. After crosslinking, the characteristic resonance peak undergoes a visible sharpening, displaying a blue shift (from 430 to 410 nm) compared with the non-crosslinked conditions. Even in this case, such behavior can be ascribed to the change in the surrounding environment due to the formation of ester bonds (during the crosslinking phase) and the consequent decrease in the pH of the films [50,51].

The MC-H/AgNPs samples were immersed in two distinct buffers (pH = 4 and 12) at 37 °C to investigate the possibility of a pH-triggered release of the nanoparticles. After 1 h, UV–vis measurements (Figure 7C) reveal silver colloids’ characteristic plasmon resonance peak at 410 nm at pH = 12. Conversely, no peak is detected at pH 4, suggesting no (or not detectable) release of AgNPs. A selective Ag release (Figure 7D), ~10 times higher at pH 12 (4.8 ± 0.6 mg_Ag_/g_MC_) than at pH 4 (0.5 ± 0.3 mg_Ag_/g_MC_), was quantified via ICP analyses. To better elucidate the mechanism underlying the pH-triggered AgNPs release, the theoretical physical parameters describing MC-H hydrogels microstructure (at the different pH values) were calculated by applying a simplified version of the Flory–Rehner model [19,20]. The average molecular weight between crosslinks ($\bar{M_{C}}$), the crosslinking density (*ρ*_c_), and the mesh size (ξ) were calculated (Supplementary Materials Table S1). In particular, the obtained ξ values were 4.09 ± 0.06, 4.33 ± 0.14, and 19.05 ± 3.07 nm for pH = 4, 7, and 12, respectively. Interestingly, the specimens’ mesh size at pH = 4 and 7 is smaller than the NPs’ diameter (12.2 ± 2.8 nm), while it is larger than the NPs diameter at pH = 12. These calculations shed new light on the selective (i.e., pH-triggered) NPs released from MC-H hydrogels. The AgNPs are physically entrapped in MC-H hydrogel mesh at low pHs, resulting in limited AgNPs release. At pH = 12, alkaline hydrolysis of ester bonds leads to hydrogel network expansion and release of AgNPs.

These results proved the possibility of controlling the release of AgNPs through an alkaline pH trigger. No studies evaluating the pH-triggered release of AgNPs from MC hydrogels were previously reported. Similar results have been obtained by Haidari H. et al., who showed the potential of selective release of AgNPs, at alkaline pH, from pH-responsive poly(mAA-co-AAm) hydrogels [12]. However, Haidari and co-workers achieved AgNPs loading by swelling the dry hydrogels in an AgNPs suspension after hydrogel synthesis and purification (necessary to remove toxic residual acrylamide monomers). Conversely, MC/AgNPs hydrogels were obtained via a one-step in situ synthesis process.

Overall, these outcomes are promising in light of the increasing concern about the possible impact of AgNPs misuse on human health [3,12]. Cytotoxic effects of AgNPs have been documented in various cell lines in vitro and are dependent on different factors (e.g., size, shape, dose, cell type), among which dosage is considered a significant parameter to be controlled by [52]. Additionally, it has been evidenced that there is a significant transdermal penetration of AgNPs into capillaries after dressings, textiles, and cosmetics [52]. In this regard, in vivo biodistribution studies have revealed how, following the dermal route, Ag translocation, accumulation, and toxicity can occur to various organs (e.g., spleen and liver) [52].

MC-H/AgNPs hydrogels prepared in this work can open the floodgates to the development of responsive systems capable of releasing AgNPs only at the occurrence of a pathological trigger (i.e., alkaline pH) in the wound milieu, avoiding the drawbacks associated with the misuse of AgNPs and thus representing a significant advantage compared to the current treatments. Further characterizations are undoubtedly needed to corroborate the claim of producing a pH-responsive platform for treating infections in chronic wounds. In particular, in vitro, antibacterial, and cytotoxicity tests could represent the first step in this direction. On this topic, Maity et al. [38] reported how MC/AgNPs nanocomposites exhibited strong antibacterial activity against several bacterial strains (i.e., *B. subtilis*, *B. cereus*, *P. aeruginosa*, *S. aureus*, and *E. coli*). Since the same AgNO_3_ concentration was used to obtain MC-H/AgNPs hydrogels in the present study, an antibacterial efficacy comparable to the one obtained by Maity et al. [38] can be expected. Regarding the in vitro cytotoxicity assessment, the non-cytotoxic nature of MC-H specimens has already been demonstrated on L929 fibroblast cells [20], also corroborating the finding of the non-toxicity of CA for amounts up to 20% *w*/w (w_CA_/w_polymer_) [53,54]. However, the cytotoxicity of MC-H/AgNPs specimens remains to be assessed. In this regard, tuning the AgNPs concentration could be necessary to achieve a trade-off between cytotoxicity and antibacterial activity [55].

## 3. Conclusions

In this work, citric acid crosslinked methylcellulose films were prepared and comprehensively characterized to develop pH-responsive systems helpful in treating infected chronic wounds. Highly crosslinked hydrogels (MC-H) showed a remarkable pH-responsive behavior based on selective hydrolysis in an alkaline environment. MC-H hydrogels were then disclosed as promising for the in situ synthesis of AgNPs and their subsequent pH-triggered delivery. Such a platform lends itself to the selective control of the proliferation of pathogens in infected chronic wounds characterized by an alkaline environment. Given that further characterizations are needed, such systems could be potentially exploited to reduce the adverse effects on host cells and tissues due to the uncontrolled AgNPs release.

## 4. Materials and Methods

### 4.1. Chemicals and Instruments

All chemicals were purchased from Sigma-Aldrich (Milan, Italy) and were used as received without further purification unless stated.

### 4.2. MC Hydrogels Preparation

Methylcellulose hydrogels were obtained according the method discussed in a previous work [19]. Citric acid (CA) was added to an MC solution (8% *w*/*v* MC in 50 mM Na_2_SO_4_) in different amounts ([CA] = 1, 3, or 5% w_CA_/w_MC_): 15 mL of solution were cast into Petri dishes (Ø = 90 mm) and oven-dried (T = 50 °C, t = 24 h). Dry films were peeled off from the Petri dishes and crosslinked by tuning the crosslinker concentration ([CA]), the crosslinking time (t_XL_) and temperature (T_XL_) [19]. Three differently crosslinked MC hydrogels were hence obtained: MC-L ([CA] = 1%, T_XL_ = 165 °C, t_XL_ = 1 min), MC-M ([CA] = 3%, T_XL_ = 178 °C, t_XL_ = 8 min), and MC-H ([CA] = 5%, T_XL_ = 190 °C, t_XL_ = 15 min), meaning low, medium, and high crosslinking degree (XLD). Non-crosslinked MC was used as a control.

### 4.3. Synthesis of MC/AgNPs Composites

The synthesis of MC/AgNPs composites was achieved by adapting a previously reported method [38]. An MC solution (0.5% *w/v*) was prepared by dispersing MC powder in hot (55 °C) distilled water. The obtained solution was cooled to room temperature under stirring, then stored at 4 °C for 24 h. The pH of the solution was adjusted to 10 using a NaOH (1 M) solution. Cold aqueous AgNO_3_ (10^−2^ M) solution was added dropwise to the MC solution in a conical flask under mild (200 rpm) stirring, achieving a final concentration of 1.11 × 10^−4^ mol_AgNO3_/g_MC_ [38]. The flask was kept in the dark, under stirring, for 24 h. The pH of the solution was then adjusted to the initial value (6.2–6.4) using an H_2_SO_4_ (0.1 M) solution. Afterward, the final SO_4_^2−^ concentration of 50 mM (i.e., the same as MC-H samples) was achieved by adding Na_2_SO_4_. CA (5% w_CA_/w_MC_) was added as a crosslinker. Lastly, 15 mL of the obtained solution were cast into Petri dishes (Ø = 90 mm) and oven-dried (T = 50 °C, t = 24 h). Dry films were first peeled off the Petri dish and then crosslinked (15 min, 190 °C). The obtained samples will be referred to as MC-H/AgNPs.

### 4.4. Swelling and Degradation Tests

The water absorption of CA-crosslinked MC hydrogels was evaluated by swelling tests in Normal Saline Solution (NSS, 9 g L^−1^ NaCl) at different pHs (4, 7, 12) and different temperatures (25, 37, 50 °C) up to 28 days. The swelling solutions’ pH was adjusted by adding a few drops of 1 M HCl or 1 M NaOH solutions. A fixed immersion ratio (MC: NSS = 20 mg: 12.5 mL) was chosen to keep the pH stable up to 72 h (the NSS solution was refreshed every 72 h). MC dry specimens were first weighted ($w_{o}$), then incubated at the test temperature (25, 37, or 50 °C) in NSS at different pH. At selected time points, the specimens were weighted ($w_{t,s}$: swollen weight at time t), and the swelling ratio (SW) was calculated as in equation (Equation (1)):

(1) $$SW\left(\%\right)=\frac{w_{t,s}-w_{o}}{w_{0}}*100$$

The stability of CA-crosslinked MC hydrogels was evaluated through degradation tests. MC dry specimens ($w_{0}$) were incubated at the test temperature (25, 37, or 50 °C) in NSS at different pH (4, 7, 12) containing 0.02% (*w/v*) NaN_3_ to prevent microbial contamination. The specimens were retrieved from the solution at selected time points, dried (50 °C, 24 h), and weighted ($w_{t,d}$: dry weight at time t). The solid gel fraction ($G_{f}$) was then calculated [20] according to equation (Equation (2)):

(2) $$G_{f}\left(\%\right)=\frac{w_{t,d}}{w_{0}}*100$$

### 4.5. MC-H Gels Microstructure: Theoretical Physical Parameters

The theoretical physical parameters describing MC-H gels microstructure were calculated according to a simplified version of the Flory–Rehner model [19,20,56,57,58]. Average molecular weight between crosslinking points ($\bar{M_{C}}$), crosslinking density ($\rho_{C}$), and mesh size (ξ) were calculated at different pH values (4, 7, and 12) at 37 °C.

The average molecular weight between crosslinking points ($\bar{M_{C}}$) was calculated using the following equations (Equations (3)–(5)) [19,57,58,59]:

(3) $$Q_{v}^{5/3}≅\frac{\bar{v}\bar{M_{C}}}{v_{l}}\left(\frac{1}{2}-\chi \right)$$

(4) $$Q_{v}=1+\left(\frac{\rho_{p}}{\rho_{s}}\left(Q_{w}-1\right)\right)$$

(5) $$Q_{w}=\frac{w_{s}}{w_{d}}$$

where $w_{s}$ and $w_{d}$ are the weights of the MC-H samples in swollen (t = 24 h) and dry conditions (t = 0), respectively. $Q_{w}$ and $Q_{v}$ represent the equilibrium weight swelling ratio and the volumetric swelling ratio, respectively.

The crosslinking density ($\rho_{C}$) was calculated according to the following equation (Equation (6)) [19,57,58]:

(6) $$\rho_{C}=\frac{1}{\bar{v}\bar{M_{C}}}$$

The mesh size (ξ) of the hydrogel at the swelling equilibrium was calculated using the following equation (Equation (7)) [19,57,58,60]:

(7) $$\xi =0.217\sqrt{\bar{M_{C}}}Q_{v}^{1/3}$$

The constant terms used in the equations (Equations (3)–(7)) are [19,20,58]:

$\rho_{p}$ = 0.276 g cm^−3^ (density of dry polymer)
$\rho_{s}$ = 1 g cm^−3^ (density of the solvent)
$\bar{v}=\frac{1}{\rho_{p}}$ = 3.623 cm^3^ g^−1^ (specific volume of dry polymer)
$v_{l}$ = 18 mol cm^−3^ (molar volume of the solvent)
χ = 0.473 (Flory polymer-solvent interaction parameter)

### 4.6. Rheological Characterization

The rheological properties of CA-crosslinked MC hydrogels at the swelling equilibrium (24 h in NSS solutions at 37 °C) were tested using a rotational rheometer (MCR 302, Anton Paar) equipped with a parallel plate geometry (Ø = 25 mm, working gap = 1 mm). Strain sweep tests were preliminarily performed on each MC hydrogel formulation to identify the linear viscoelastic region (LVR) by applying an oscillatory strain in the 0.01–10% strain range (γ) at 37 °C and 1 Hz frequency (ν). Then, to determine the transition temperature (T_t_) of each specimen, temperature sweep tests were performed in the 10–60 °C range, applying a temperature ramp of 2 °C min^−1^, γ = 0.1% (i.e., LVR obtained by strain sweep tests), ν = 1 Hz frequency. For each hydrogel composition, the T_t_ was identified from storage modulus (G′) and complex viscosity (η*) curves. Briefly, from each curve, the T_t_ was determined as the intersection between the interpolant of the initial T range (T < T_t_) and the interpolant of the following T range (T > T_t_) [27,28].

### 4.7. ^1^H-NMR Characterization

The MC-H formulation was considered the most promising for this work due to its remarkable pH-responsiveness. The pH responsivity of MC-H samples was further investigated using ^1^H-NMR spectrometry. ^1^H-NMR spectra were collected using a Bruker ARX 400 spectrometer (400 MHz ^1^H resonance frequency). All spectra were recorded in D_2_O containing 0.05% (*w/w*) of 3-(trimethylsilyl)propionic 2,2,3,3,-d_4_ acid sodium salt as internal standard. Chemical shifts (d, ppm) are reported relative to the internal standard.

Explorative analyses were carried out on washed MC-H samples. Removal of unreacted CA from the MC-H specimens was achieved by rinsing MC-H samples several times in distilled water until reaching neutral pH [19]. The ^1^H-NMR spectra of washed MC-H samples were then collected in the following conditions: (i) at the swelling equilibrium (20 mg MC-H in 0.7 mL D_2_O for 24 h), (ii) after the addition of 35 µL NaOD (30 wt.% in D_2_O), (iii) after the addition of further 35 µL NaOD, and (iv) 24 h after the addition of 70 µL NaOD. All measurements were carried out at 30 °C.

Then, the ^1^H-NMR spectra of non-washed MC-H specimens were collected to retrace the swelling and degradation tests. Briefly, MC-H were swollen in NSS at different pH (4, 7 and 12) for 24 h (i.e., swelling equilibrium), keeping fixed the immersion ratio (MC-H: NSS = 20 mg: 12.5 mL). Then, the samples were freeze-dried. After lyophilization, each specimen was swollen in 1 mL of D_2_O for 24 h before ^1^H-NMR spectra collection. For each pH condition, ^1^H-NMR spectra were collected at 30, 37, and 50 °C.

### 4.8. TEM Characterization

The shape, size, and distribution of silver nanoparticles in the MC/AgNPs solutions were assessed by transmission electron microscopy (TEM, Philips CM200-FEG) at an accelerating voltage of 200 kV. A drop of the sample (after 1:10 dilution in dH_2_O) was withdrawn, deposited on a carbon-coated copper net (mesh size 200), and air-dried overnight at room temperature. To estimate the shape and size of silver nanoparticles, 50 images were analyzed by ImageJ software (ImageJ, v. 1.53, NIH). Prism 8 (GraphPad Software, La Jolla, CA, USA) was then used for the particle size distribution analysis.

### 4.9. UV–Vis Characterization

UV–vis spectroscopy (Synergy H1 spectrophotometer, BioTek) was used to monitor the intensity of the Localized Surface Plasmon Resonance (LSPR) absorption band of silver nanoparticles in the MC samples. UV–vis studies were performed (i) on MC/AgNPs solutions, (ii) on MC-H/AgNPs dry films, and (iii) on the swelling solutions of MC-H/AgNPs specimens. In the latter case, UV–vis measurements were carried out to assess the pH-triggered AgNPs release from MC-H/AgNPs specimens. To do this, MC-H/AgNPs specimens were first briefly rinsed with dH_2_O to remove AgNPs not embedded in the film. Then, the specimens were immersed in 10 mM acetate (pH = 4) or phosphate (pH = 12) buffers, incubated at 37 °C for 1 h, and the swelling media were analyzed. Spectra were acquired in the wavelength range 250–700 nm, with a resolution of 1 nm. The solutions were read using a Take3 Micro-Volume Plate support (BioTek), while dry films used a Slide adapter (BioTek, 1220548).

### 4.10. ICP Analysis

To quantify the amount of Ag released by MC-H/AgNPs specimens as a function of the pH, the samples were first briefly rinsed with dH_2_O to remove superficial AgNPs. Then, the specimens were immersed in 10 mM acetate (pH = 4) or phosphate (pH = 12) buffers and incubated at 37 °C (pH 7 was not investigated based on the outcomes of physicochemical and rheological characterization). After 1 h, the eluates were withdrawn and analyzed by inductively coupled plasma optical emission spectrometry (ICP-OES).

### 4.11. Data Analysis

Unless stated, the tests were run in triplicate (*n* = 3), and data are expressed as mean ± standard deviation (SD). Statistical analysis was performed by Prism 8 (GraphPad Software, La Jolla, CA, USA). Comparisons among the groups were performed by ANOVA (one-way or two-way), and results of *p* < 0.05 were considered statistically significant.

## Figures and Tables

**Table 1:** T_t_ calculated from temperature sweep tests for each MC hydrogel formulation. * = *p* < 0.05 compared with MC control.

	MC	MC-L	MC-M	MC-H
pH = 4	36.5 ± 0.7	32.8 ± 0.4 *	34.0 ± 0.7	-
pH = 7	36.8 ± 0.4	33.5 ± 0.7	35.0 ± 1.4	-
pH = 12	33.5 ± 4.9	26.5 ± 0.7	30.5 ± 2.1	34.8 ± 2.5

## Data Availability

Not applicable.

## Supplementary Materials

The following supporting information can be downloaded at: https://www.mdpi.com/article/10.3390/gels8050298/s1, Supplementary Materials S1: Swelling and gel fraction; Supplementary Materials S2: Flory–Rehner model; Supplementary Materials S3: Rheology; Supplementary Materials S4: ^1^H-NMR.
