# Supplementary material for: Smart Methylcellulose Hydrogels for pH-Triggered Delivery of Silver Nanoparticles
Source: Gels. 2022 May 12;8(5):298. doi: 10.3390/gels8050298 (PMC9140787; doi:10.3390/gels8050298)
Supplement: Supplementary file 1 [file gels-08-00298-s001.zip › gels-1702218-supplementary.pdf]

gels

Supplementary Materials

Smart Methylcellulose Hydrogels for pH-Triggered Delivery of Silver Nanoparticles

Lorenzo Bonetti [1,*], Andrea Fiorati [1,2,] Agnese D'Agostino [1,2], Carlo Maria Pelacani [1], Roberto Chiesa [1,2], Silvia Farè [1,2] and Luigi De Nardo [1,2]

Supplementary Materials S1: Swelling and gel fraction

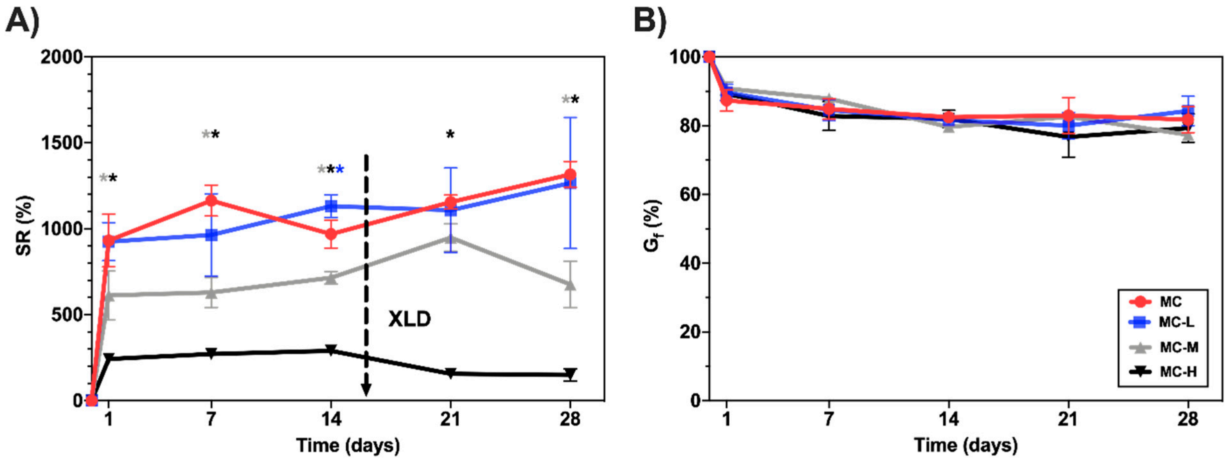

Figure S1. (**A**) Swelling ratio (SR %) vs. time of MC hydrogels in NSS at T = 37 °C and pH = 7. (**B**) Gel fraction (G_f %) vs. time curves of MC hydrogels samples in NSS at T = 37 °C and pH = 7. * = *p* < 0.05 compared to MC control (* = MC vs. MC-L, * = MC vs. MC-M, * = MC vs. MC-H).

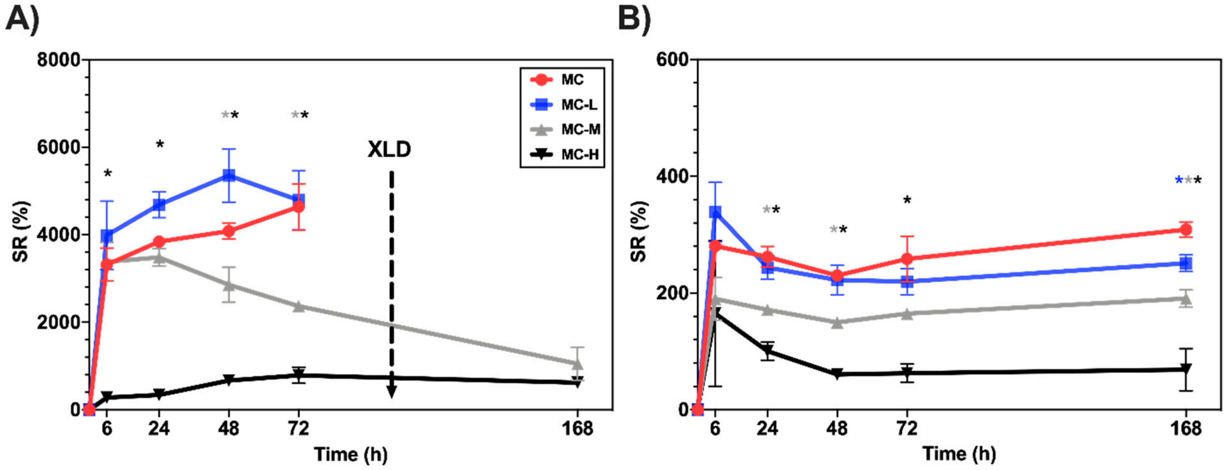

Figure S2. Swelling ratio (SR %) vs. time of MC hydrogels in NSS (**A**) at T = 25 °C and pH = 7, and (**B**) at T = 50 °C and pH = 7. * = p < 0.05 compared to MC control. * = *p* < 0.05 compared to MC control (* = MC vs. MC-L, * = MC vs. MC-M, * = MC vs. MC-H).

Supplementary Materials S2: Flory-Rehner model

Table S1 reports the theoretical physical parameters calculated for MC-H samples at the different pH levels (4, 7, 12), at 37 °C.

Table S1. – Theoretical physical parameters describing MC-H microstructure at the different pH levels (4, 7, 12) at 37 °C. The symbols indicate statistical differences ($p < 0.05$) between the groups.

Parameter	pH = 4	pH = 7	pH = 12
$\overline{M_C}$ (g mol^{-1}) · 10^{2}	$2.94 \pm 0.06^{*}$	$3.20 \pm 0.15^{\#}$	$26.6 \pm 6.2^{*\#}$
ρ_C (mol cm^{-3}) · 10^{-4}	$9.39 \pm 0.20^{\dagger}$	$8.65 \pm 0.40^{¥}$	$1.07 \pm 0.22^{\dagger ¥}$
ξ (nm)	4.09 ± 0.06^{f}	$4.33 \pm 0.14^{€}$	$19.05 \pm 3.07^{f€}$

Supplementary Materials S3: Rheology

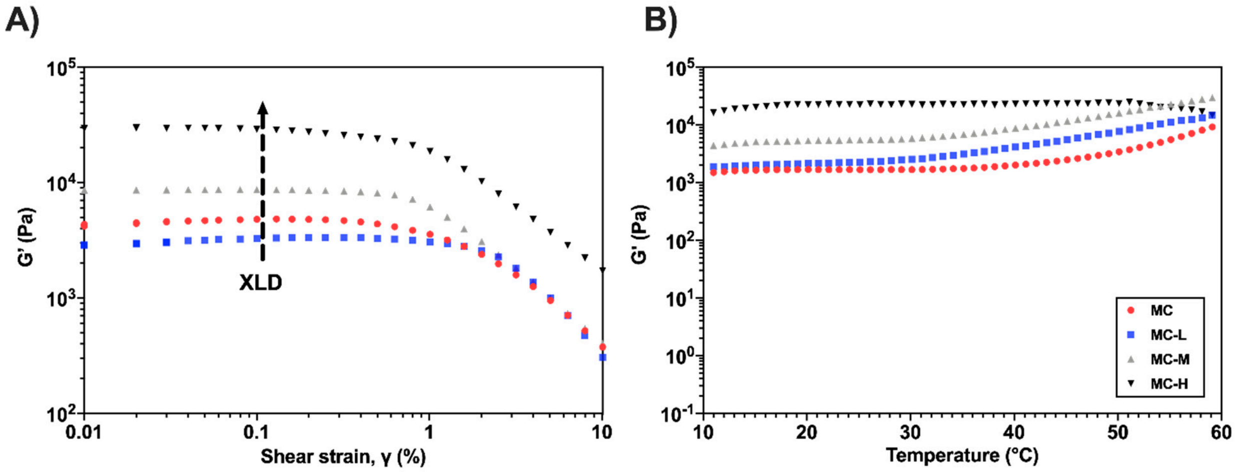

Figure S3. (**A**) Representative G′ vs. γ curves for strain sweep tests on MC samples at pH = 7. (**B**) Representative G′ vs. T curves for temperature sweep tests on MC samples at pH = 7.

Supplementary Materials S4: ^{1}H-NMR.

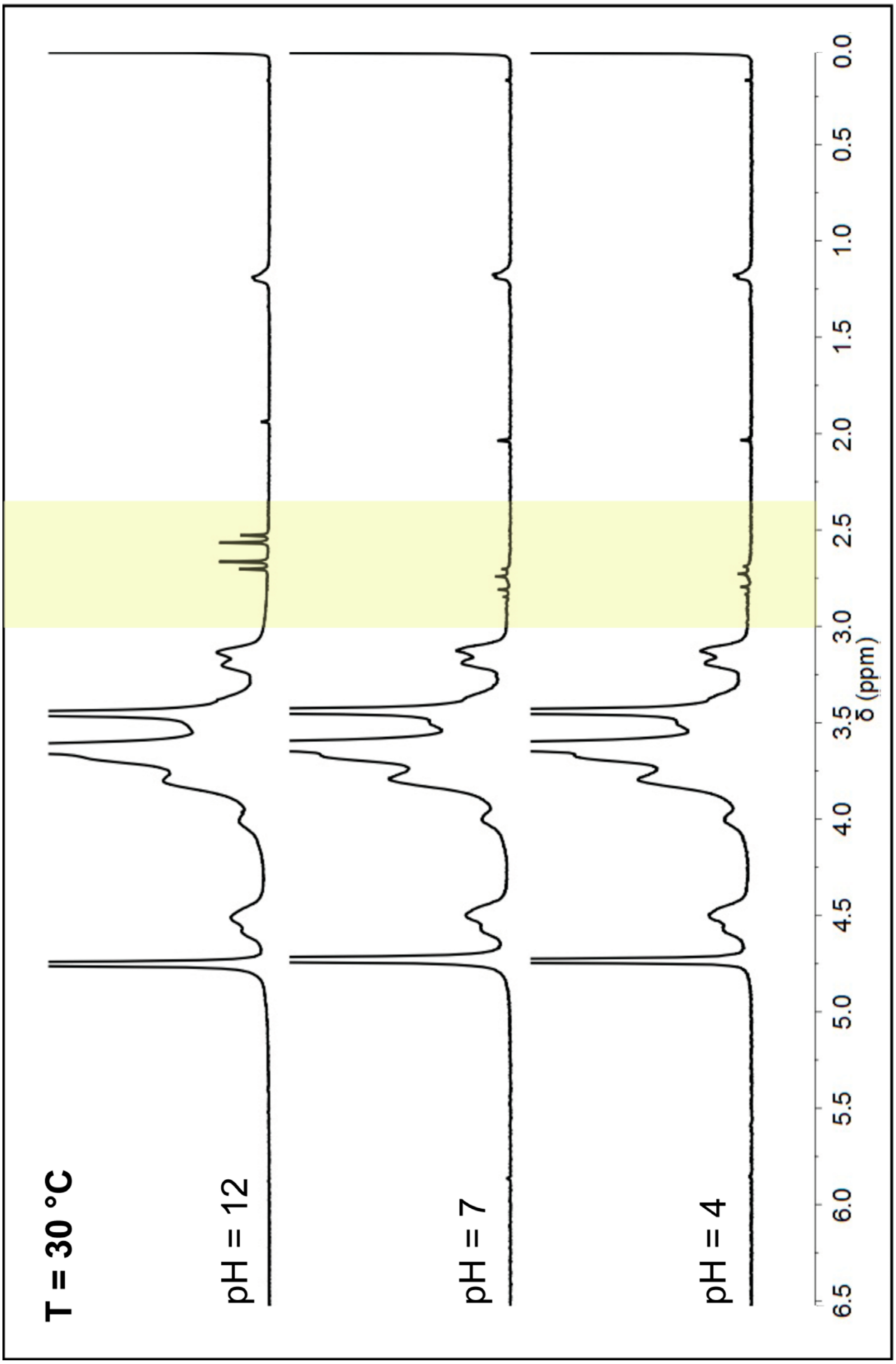

Figure S4. ^{1}H-NMR spectrum of non-washed MC-H specimens swelled at different pH (4, 7, and 12). All the spectra were acquired at 30 °C. The yellow area highlights the $-CH_2$ peaks of CA.

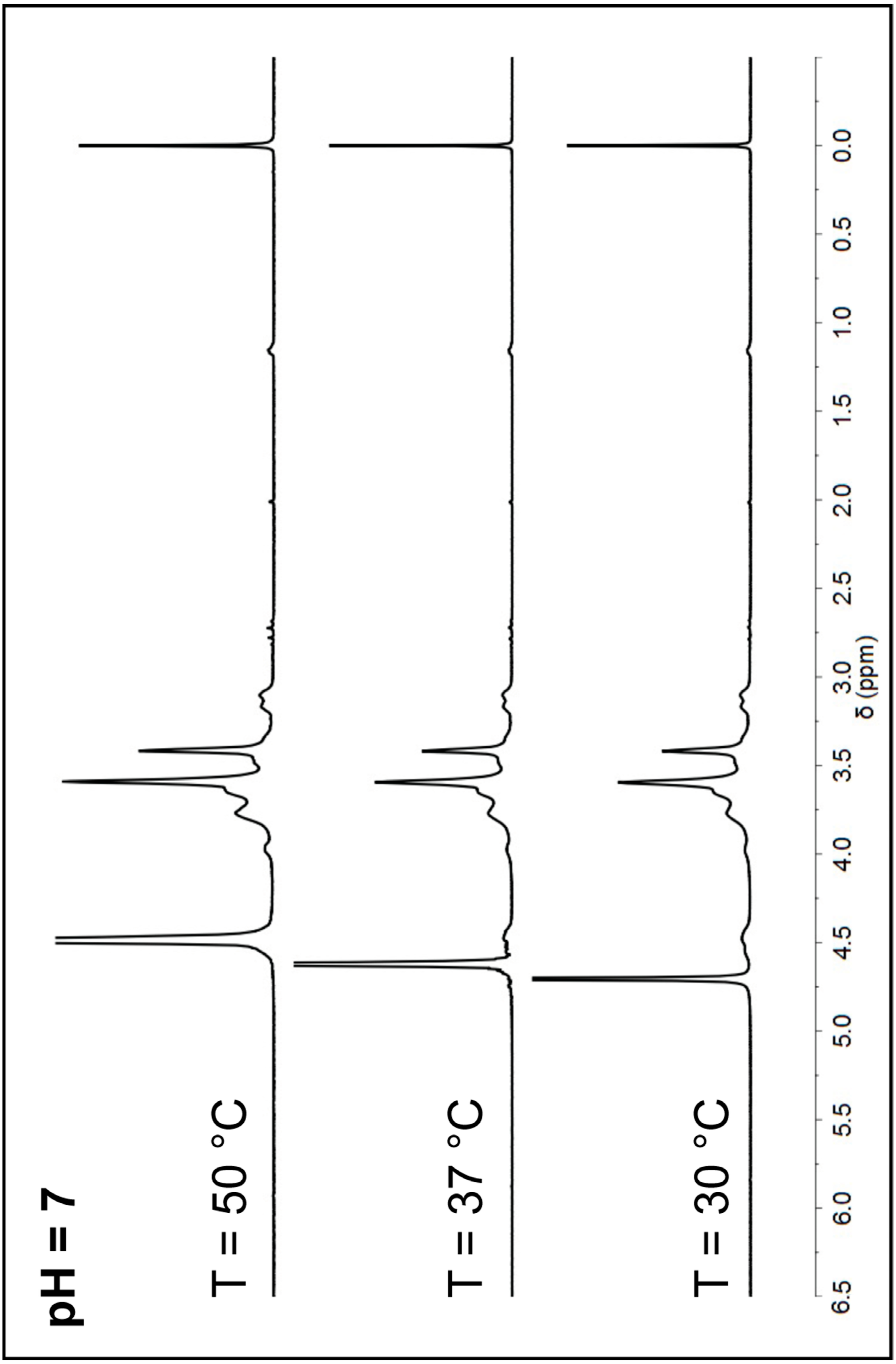

Figure S5. ^{1}H-NMR spectrum of MC-H swelled at pH = 7 at different temperatures (30, 37, and 50 °C).
